# Trimetazidine and allopurinol for the prevention of Contrast-associated acute kidney injury in elective percutaneous coronary intervention: a randomized controlled trial

**DOI:** 10.1007/s00228-025-03985-6

**Published:** 2026-01-22

**Authors:** Nourhan Osama Ali, Naglaa Samir Bazan, Hatem Hossam Mowafy, Mohamed E.A. Abdelrahim, Hadeer S. Harb

**Affiliations:** 1 Egyptian Drug Authority (EDA), Cairo, Egypt; 2 Critical Care Medicine Department, Kasr Al Ainy Hospitals, Cairo University, Cairo, Egypt; 3 Clinical Pharmacy Department, College of Pharmacy, King Khalid University, Abha, Saudi Arabia; 4 Clinical Pharmacy Department, Faculty of Pharmacy, Beni-Suef University, Beni-Suef, Egypt

**Keywords:** Contrast-associated acute kidney injury, Percutaneous coronary intervention, Contrast volume, Acute kidney injury, SGLT-2 inhibitors, DPP-4 inhibitors

## Abstract

**Purpose:**

Contrast-associated acute kidney injury (CA-AKI) is a significant concern following percutaneous coronary intervention (PCI). This study assessed whether adding trimetazidine (TMZ) or TMZ/allopurinol to standard hydration reduces CA-AKI among elective PCI patients.

**Methods:**

129 patients undergoing elective PCI were randomized into three groups: Group 1 received (IV isotonic saline + TMZ + allopurinol), Group 2 received (IV isotonic saline + TMZ), and Group 3 (control) received (IV isotonic saline only). The primary outcome was the incidence of CA-AKI at 24- and 48-hour post-PCI. Risk was stratified using the Mehran and the Age, Creatinine, and Ejection Fraction (ACEF) scores.

**Results:**

Group 1 demonstrated a non-significant reduction in CA-AKI incidence compared with Groups 2 and 3 (24 h: 4.65%, 4.55%, and 9.52%; 48 h: 16.28%, 20.45%, and 28.57%; *p* > 0.05). All patients were classified as low–moderate risk by ACEF and Mehran scores, neither of which predicted CA-AKI. At 48 h, SGLT2 inhibitors users demonstrated a smaller rise in creatinine compared with non-users (–0.04 ± 0.158 mg/dL vs. 0.096 ± 0.237 mg/dL, *p* < 0.05), as did DPP-4 inhibitor users (–0.05 ± 0.217 mg/dL vs. +0.098 ± 0.237 mg/dL, *p* < 0.05). Diuretics were associated with greater increases (*p* < 0.05).

**Conclusion:**

The TMZ–allopurinol combination showed a favorable but non-significant trend toward reducing CA-AKI, while ACEF and Mehran scores demonstrated limited predictive value. Improved risk-stratification tools and larger studies in higher-risk patients are needed. SGLT2 and DPP-4 inhibitors showed smaller creatinine increases, suggesting possible nephroprotection, but this remains exploratory.

## Introduction

Iodine contrast media (ICM) have been associated with acute kidney injury (AKI), historically termed contrast-induced nephropathy (CIN), occurring in 11–40% of exposed patients [1]. The term CIN has been replaced by contrast-induced AKI (CI-AKI) in Kidney Disease: Improving Global Outcomes (KDIGO) guidelines [2].The American College of Radiology (ACR) [3]and the European Society of Urogenital Radiology (ESUR) [4] further introduced the terms contrast-associated (CA-AKI) and post-contrast (PC-AKI) acute kidney injury. CA-AKI refers to any AKI occurring within 48 h of contrast media (CM) exposure, and this terminology is used throughout this manuscript [1]. Renal function typically declines transiently after ICM exposure, peaking within 2–3 days and returning to normal in 1–3 weeks. However, CA-AKI has been linked to increased mortality, morbidity, and longer hospital stays [5, 6]. Risk factors include older age, baseline renal insufficiency, hyperuricemia, diabetes, medication-related contributors, and the kind and volume of CM [7]. Its incidence is notably higher after percutaneous coronary intervention (PCI), contributing to adverse short- and long-term outcomes [8]. The Mehran score, incorporating eight clinical and procedural criteria, and the Age, Creatinine, and Ejection Fraction (ACEF) score are widely used to estimate CA-AKI risk in PCI populations [9–11].

All CM are potentially nephrotoxic, primarily through vascular constriction and inhibition of tubule glomerular feedback, oxidative stress, and direct tubular epithelial injury. Non-ionic low-osmolar and iso-osmolar agents (LOCM and IOCM) are considered less nephrotoxic than the high osmolar agents (HOCM) [12]. International guidelines are consistent in recommending intravenous (IV) hydration using isotonic saline as the main strategy to prevent CA-AKI. KDIGO advises IV hydration and discourages oral fluids alone or prophylactic dialysis [2]. ESUR confirms the effectiveness of isotonic saline or bicarbonate and notes that no pharmacologic agent, including N-acetylcysteine, statins, vitamin C, or trimetazidine, has shown consistent benefit, and they do not support prophylactic renal replacement therapy [4]. European Society of Cardiology (ESC) and the European Association for Cardio-Thoracic Surgery (EACTS) guideline similarly endorse hydration and low- or iso-osmolar agents while advising against N-acetylcysteine or bicarbonate [13].The ACR Manual on Contrast Media (2025) emphasizes that IV hydration is the only preventive strategy supported by consistent evidence [14]. These recommendations highlight the need to explore potential adjunctive therapies such as trimetazidine and allopurinol.

Allopurinol has been proposed as a strategy for preventing AKI during coronary angiography through xanthine oxidase inhibition, reducing oxidative stress and uric acid levels [15–18]. Although it demonstrates renoprotective effects in ischemia models, clinical findings remain inconsistent, and recent meta-analyses recommend evaluating allopurinol in combination with other agents [18–21]. Trimetazidine (TMZ), a cellular anti-ischemic agent, reduces intracellular acidosis, limits the peroxidation of membrane lipids, prevents neutrophil infiltration following ischemia-reperfusion, avoids excessive release of oxygen-free radicals, changes energy metabolism from fatty acid oxidation to glucose oxidation, and maintains adenosine triphosphate stores [22]. However, evidence for TMZ in CA-AKI prevention remains mixed [23–26]. A trial comparing TMZ and allopurinol found no significant benefit, possibly due to the inclusion of predominantly low-risk patients [24].

Consequently, there is insufficient evidence to recommend TMZ or allopurinol, alone or in combination, for CA-AKI prevention beyond standard hydration in patients with normal glomerular filtration rates and varied risk profiles, and their potential synergistic effects remain unstudied. Therefore, this study evaluated the effectiveness of IV hydration combined with TMZ or TMZ/allopurinol compared with hydration alone in preventing CA-AKI among patients undergoing elective PCI.

## Materials and methods

### Study design

Prospective, randomized, controlled, open-label study.

#### Study population

Patients undergoing elective PCI in the angiography unit at the Critical Care Medicine Department, Cairo University Hospitals, from July 2022 to February 2024 were screened for inclusion in the study. In July 2022, Cairo University’s faculty of medicine’s ethical committee accepted the study, assigning it the ethical number S-2-2022. Written informed consent was provided by each patient. The trial was identified by its registration number (NCT05540184) on ClinicalTrials.gov.

#### Inclusion criteria [19]

Only patients undergoing elective PCI were eligible for inclusion. All patients with GFR > 60 ml/min/1.73 m² aged 18–80 years planned to undergo elective PCI with a low, moderate, or high risk of CA-AKI based on the Mehran risk score were included [9].

#### Exclusion Criteria [9]

Patients requiring emergency or primary PCI (e.g., for acute coronary syndromes or hemodynamic instability) were excluded from screening. Patients with a history of allopurinol intake, hepatic failure, pregnancy or lactation, AKI defined according to KDIGO 2012 criteria (an increase in serum creatinine by ≥ 0.3 mg/dL within 48 h, an increase to ≥ 1.5 times baseline within the prior 7 days, or urine output < 0.5 mL/kg/h for 6 h; patients meeting any of these criteria were excluded, regardless of AKI stage), renal insufficiency (eGFR < 60 mL/min/1.73 m², since advanced CKD is associated with a markedly higher baseline risk of CA-AKI and increased susceptibility to drug accumulation, which could compromise patient safety, raise ethical concerns, and obscure the preventive effect of the study medications [2, 4, 14], gout (serum uric acid > 10 mg/dL), any nephrotoxic drug intake within 48 h before the procedure (such as NSAIDs, aminoglycosides, amphotericin B, cisplatin, or other chemotherapeutic agents), pulmonary edema, cardiogenic shock, and requirement for mechanical ventilation. In line with ESUR guidelines, ACE inhibitors and angiotensin receptor blockers were not discontinued before contrast medium administration. However, according to hospital protocol, Metformin was withheld from the time of contrast medium administration and restarted within 48 h if renal function remained stable. Patients with serum uric acid > 10 mg/dL were excluded to avoid confounding from uncontrolled hyperuricemia or gout, which requires separate urate-lowering therapy. Furthermore, because initiation of allopurinol during an acute gout attack may precipitate further flares, only patients with stable uric acid levels were eligible for inclusion [21, 27].

#### Randomization

Patients were randomly assigned into 3 groups as follows:

##### Group 1 (TMZ + Allopurinol, *n* = 43)

In addition to hydration, patients received 300 mg of allopurinol once daily five hours before the procedure and on the day after, along with 35 mg of TMZ once daily before and up to 24 h after the procedure.

##### Group 2 (TMZ only, *n* = 44)

In addition to hydration, patients received 35 mg of TMZ once daily before and up to 24 h after the procedure.

##### Group 3 (Control, *n* = 42)

Patients received hydration only [23, 28]. Using IV normal saline (0.9% sodium chloride) at a rate of 1 mL/kg/day, 3 to 4 h before the procedure and up to 24 h after the procedure, with a maximum rate of 100 mL/hr, all patients were hydrated both before and after PCI [23].

Before PCI, all patients were given either 300 mg of aspirin and 600 mg of clopidogrel or 300 mg of aspirin and 180 mg of ticagrelor. Using an online randomization tool, eligible patients were randomized by block to the three groups [29].Patients were randomized using sequentially numbered, opaque sealed envelopes (SNOSE) to establish allocation concealment [30]. Block randomization with a fixed block size of six was used to ensure balanced allocation across the three study groups (Control, TMZ, and TMZ + Allopurinol) in a 1:1:1 ratio.

#### Hydration protocol

All patients received intravenous (IV) hydration with normal saline (0.9% sodium chloride) at a rate of 1 mL/kg/hour starting 3–4 h before and continued up to 24 h after PCI, with a maximum rate of 100 mL/hour. According to hospital protocol, patients with left ventricular systolic dysfunction (EF < 50%) did not routinely receive IV hydration because of the risk of fluid overload and pulmonary edema. In these patients, preventive measures included minimizing contrast volume, using iso- or low-osmolar contrast media, and close hemodynamic and renal function monitoring. This individualized approach aligns with the ESUR Contrast Medium Safety Committee recommendations, which advise cautious, clinician-guided hydration in patients with advanced heart failure (NYHA class III–IV) or end-stage renal disease (CKD stage V) (Level of Evidence D) [4].

#### Contrast media and data collection

CM used were HOCM (Meglumine Ioxitalamate) Telebrix^®^, Amoun Pharma, or LOCM (Iohexol) Omipaque^®^, GE HealthCare. The choice of which CM type to use was determined based on expected prolonged procedures and the availability of the dye at the hospital. The volume of CM used throughout the procedure was recorded, and the maximum permitted CM volume was determined using the maximal allowable contrast dose equation (MACD = 5 × body weight/Scr) [31].

Comorbidities, current medications, and demographic information were gathered. Serum creatinine, urea, and uric acid concentrations were measured by taking baseline blood samples from patients upon admission and 24- and 48-hours following PCI. The Modification of Diet in Renal Disease (MDRD) equation [32] was used to estimate the GFR, and the Cockcroft-Gault equation was used to estimate creatinine clearance (CrCl) [33]. Furthermore, the risk of CIN stratification was computed and documented using the ACEF calculator and Mehran score.

## ACEF score and Mehran score

### Mehran definition

Nephrotoxicity following PCI was predicted using the Mehran score [9]. Hypotension, chronic heart failure, the use of an intra-aortic balloon pump, a Scr level greater than 1.5 mg/dl, age greater than 75 years, anemia, diabetes mellitus (DM), an estimated GFR (eGFR) below 60 ml/min, and CM volume were among the conditions covered. Each patient was given a weighted integer, and the sum of these constituted their risk score, which was as follows: A score of 5 or lower indicates low risk, 6–10 points: moderate risk, 11–15 points: high risk, 16: very high risk.

### ACEF score

The ACEF score includes age, Scr, and left ventricular ejection fraction. Patients were stratified into risk groups based on ACEF tertiles: low-risk (first tertile, ACEF < 1.2), moderate-risk (second tertile, ACEF 1.2–1.5), and high-risk (third tertile, ACEF > 1.5) [10].

### Primary outcome

The primary outcome was the incidence of CA-AKI defined as a relative increase in either CrCl or Scr of at least 25% above the baseline level or an absolute rise in Scr of at least 0.5 mg/dl at 24- and 48-hours following exposure to CM [34, 35].

### Secondary outcome

Changes in Scr, Urea, Uric Acid, CrCl, and eGFR 48 h post-procedure among patients depending on their Mehran and ACEF scores. In addition, risk factors associated with change in Scr were assessed [9, 10].

### Sample size calculation

The sample size was calculated based on a two-group comparison using data from Bodagh et al. (2019), which demonstrated a reduction in CA-AKI incidence from 38% in the control group to 12% with allopurinol. Assuming a similar effect size for our primary comparison, TMZ plus allopurinol versus control, a two-sided chi-square test with 80% power and α = 0.05 yielded a requirement of 42 subjects per group. To account for an anticipated 20% attrition rate, the target sample size was increased to 51 participants per group. Ultimately, 129 patients (42 in the control group, 44 in the TMZ-only group, and 43 in the combination group) were enrolled, which was slightly below the adjusted target but sufficient to detect moderate to large treatment effects. A third arm receiving TMZ alone was included to explore potential additive or synergistic effects. However, the study was not powered for all pairwise comparisons, and results involving the TMZ-alone group should be interpreted accordingly. Sample size calculations were performed using G*Power software (version 3.1.9.2, Heinrich Heine Universität Düsseldorf) [28].

### Statistical analysis

Categorical variables were expressed as frequencies (n, %) and compared using the Chi-square test. When expected cell counts were low, the Monte Carlo simulation method (§) was applied to obtain more accurate p-values. For continuous variables that were not normally distributed, values were summarized as medians with interquartile ranges (IQR) and compared using the Kruskal–Wallis test (¥). For normally distributed quantitative variables, comparisons were performed using Analysis of Variance (ANOVA) when the assumption of equal variances was met; otherwise, the Welch test was applied. To identify predictors of CA-AKI at 24 and 48 h, the logistic regression analysis was conducted, with odds ratios (OR) and their 95% confidence intervals (CI) reported as measures of association. Multivariable models were fitted to adjust for potential confounding factors identified from the univariate analyses. To examine factors influencing the change in Scr levels at 24 and 48 h, linear regression models were used. All statistical analyses were performed using IBM SPSS Statistics software, version 29. A p-value < 0.05 was considered statistically significant. All analyses followed the intention-to-treat principle.

## Results

**Fig. 1:**
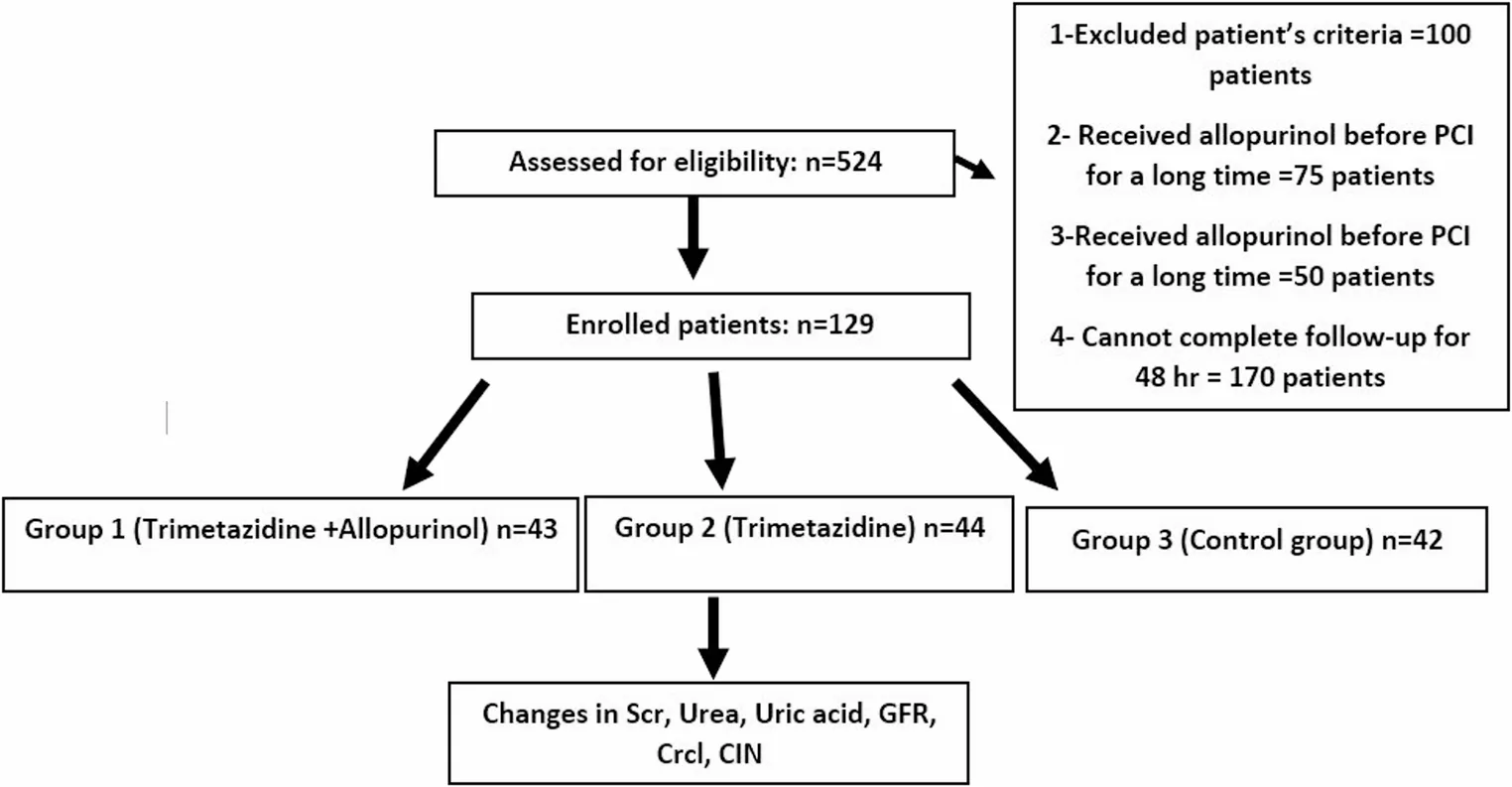
Flowchart of patient enrollment and group allocation

A total of 524 patients undergoing elective PCI in the angiography unit at the Critical Care Medicine Department, Cairo University Hospitals were screened for inclusion in the study. Of the 524 patients screened, 395 were excluded before randomization, including 170 who could not commit to the 48-hour follow-up laboratory assessment. The inclusion criteria were only met in 129 cases (Fig. 1). They were divided into three tested groups. Baseline demographic and clinical characteristics, dye volume used, and concomitant medications were similar between the three groups. Risk stratification by the Mehran and ACEF scores confirmed that all participants were low to moderate risk, with no significant differences between groups (Table 1). However, patients in Group 1 (IV isotonic saline + TMZ + allopurinol) and Group 2 (IV isotonic saline + TMZ) used the HOCM (Ioxitalamate) dye at a much higher proportion than patients in Group 3 (IV isotonic saline only), compared to the LOCM (Iohexol) dye which may represent a potential confounding variable (Table 2). Additionally, baseline uric acid in Group 1 (IV isotonic saline + TMZ + allopurinol) was significantly lower than in Group 3 (IV isotonic saline only), Table 3.

**Table 1:** Comparison of baseline characteristics across the three study groups

Groups	Control (*n* = 42)	Trimetazidine (*n* = 44)	Combination (*n* = 43)	*p*-value
Male, n (%)	26 (61.9)	30 (68.18)	20 (46.51)	0.108
Female, n (%)	16 (38.1)	14 (31.82)	23 (53.49)	
Age (Mean *±* SD), years	59.40 *±* 9.90	56.20 *±* 10.40	58.80 *±* 9.00	0.26
BMI (Mean *±* SD), Kg/m^2^	29.10 *±* 6.40	25.50 *±* 5.30	26.20 *±* 5.70	0.01*
Comorbidities, n (%)				
Obesity	13(30.95)	7 (15.91)	8 (18.60)	0.199
Diabetes	21 (50.00)	23 (52.27)	27 (62.79)	0.447
Hypertension	31 (73.81)	29 (65.91)	35 (81.40)	0.261
Hypotension	1(2.38)	0 (0.00)	0 (0.00)	0.352
Previous PCI	7 (16.67)	11 (25.00)	13 (30.23)	0.337
Anemia	21 (50.00)	16 (36.36)	11 (25.58)	0.066
Surgery	3 (7.14)	1 (2.27)	0 (0.00)	0.120
Hypothyroidism	1(2.38)	1 (2.27)	1 (2.33)	1.00
Hydration volume for patients EF < 50, n (%), ml/hr	6 (14.29)	10 (22.73)	7 (16.28)	0.56
Medications, n (%)				
B-blocker	28 (66.67)	28 (63.64)	31 (72.09)	0.696
ACE inhibitors	7 (16.67)	10 (22.73)	12 (27.91)	0.46
ARBs	12 (28.57)	8 (18.18)	8 (18.60)	0.421
CCB	3 (7.14)	6 (13.64)	3 (6.98)	0.596
Nitroglycerin	16 (38.10)	17 (38.64)	11 (25.58)	0.352
Isosorbide Dinitrate	4 (9.52)	3 (6.82)	7 (16.28)	0.404
Nicorandil	3 (7.14)	6 (13.64)	6 (13.95)	0.543
Diuretics	6 (14.29)	5 (11.36)	8 (18.60)	0.632
Ezetimibe	8 (19.05)	6 (13.64)	10 (23.26)	0.512
Insulin	9 (21.43)	7 (15.91)	10 (23.26)	0.673
DPP4 inhibitors	6 (14.29)	4 (9.09)	4 (9.30)	0.73
SGLT-2 inhibitors	3 (7.14)	6 (13.64)	5 (11.63)	0.68
Anticoagulant	2 (4.76)	3 (6.82)	1 (2.33)	0.785
Mehran and ACEF risk scores, n (%)				
Mehran moderate risk	14 (33.33)	20 (45.45)	16 (37.21)	0.498
Mehran low risk	28 (66.67)	24 (54.55)	27 (62.79)	
ACEF high risk tertile > 1.5	5 (11.90)	4 (9.09)	1 (2.33)	0.25
ACEF low risk tertile < 1.5	39 (88.1)	38 (90.91)	42 (97.67)	

Data are expressed as mean ±SD or Median (Minimum-Maximum), or n(%).

BMI, Body mass index, PCI, percutaneous coronary intervention; ACEI, angiotensin−converting enzyme inhibitor; ARB, angiotensin receptor blocker; CCBs, Calcium channel blocker; DPP−4 Inhibition, dipeptidyl peptidase 4 inhibitor; SGLT−2, sodium−glucose cotransporter−2 inhibitors

**Table 2:** Comparison of laboratory Parameters, ejection Fraction, and contrast dye type and volume across the three study groups

Groups	Control (*n* = 42)	Trimetazidine (*n* = 44)	Combination (*n* = 43)	*p*- value
Lab Investigation and Examination Parameters:				
Hemoglobin (Mean *±* SD), mg/dl	12.43 *±* 1.68	13.10 *±* 1.90	13.01 *±* 1.78	0.18
HCt (Mean *±* SD), %	37.51 *±* 5.47	39.48 *±* 5.57	39.54 *±* 5.45	0.16
LVEF, (Mean *±* SD), %	59.14 *±* 9.92	57.66 *±* 11.06	58.37 *±* 7.48	0.78
INR (Mean *±* SD)	1.09 *±* 0.18	1.05 *±* 0.16	1.03 *±* 0.15	0.21
HCV, n (%), IU/ml	4 (9.52)	1 (2.27)	2 (4.65)	0.26
HBV, n (%), IU/ml	0 (0.00)	1 (2.27)	0 (0.00)	1.00
**Contrast Dye Type and Volume**:				
Iohexol (Omnipaque), n (%)	23 (54.76)	15 (34.09)	13 (30.23)	0.046*
Sodium Ioxitalamate (Telebrix), n (%)	19 (45.24)	29 (65.91)	30 (69.77)	
Dye volume (Mean *±* SD), ml	115.48 *±* 111.80	190.91 *±* 145.98	198.84 *±* 135.62	0.01*
Maximal dose of CM, (Mean + SD), ml	492.38 *±* 126.93	462.61 *±* 97.22	467.08 *±* 109.55	0.42

Data are expressed as mean ±SD or Median (Minimum−Maximum), or n (%)

HCt, Hematocrit; INR, International normalized ratio; CM, Contrast Media; LVEF, left ventricular ejection fraction; HCV, Hepatitis C; HBV, Hepatitis B

For significances * *p*<0.05, ** *P*<0.01, *** *P*<0.001

**Table 3:** Scr, Urea, uric acid, GFR, and CrCl in the three study groups at Baseline, 24 h, and 48 h after contrast media administration

Groups	Control (*n* = 42)	Trimetazidine (*n* = 44)	Combination (*n* = 43)	*p*- value	Omega-squared (95% CI)
	Mean *±* SD	Mean *±* SD	Mean *±* SD		
Scr, (Mean **±** SD), (mg/dl)					
Baseline	0.98 *±* 0.18	0.96 *±* 0.16	0.96 *±* 0.19	0.88	−0.014(−0.016, 0.009)
24 h post PCI	0.80 *±* 0.17	0.77 *±* 0.21	0.81 *±* 0.24	0.69	−0.010(−0.016, 0.030)
48 h post PCI	1.07 *±* 0.25	1.06 *±* 0.25	1.01 *±* 0.27	0.46	−0.004(−0.016, 0.048)
Absolute changes (baseline to 48 h) post-PCI	0.10 *±* 0.25	0.10 *±* 0.22	0.05 *±* 0.23	0.50	−0.005(−0.016, 0.044)
Uric acid, (Mean **±** SD), (mg/dl)					
Baseline	5.24 *±* 1.37	5.22 *±* 1.57	4.11 *±* 1.31	< 0.001***	0.106(0.010, 0.212)
24 h post PCI	5.46 *±* 1.55	5.12 *±* 1.56	4.07 *±* 1.24	< 0.001***	0.128(0.026, 0.235)
48 h post PCI	5.37 *±* 1.62	5.46 *±* 1.56	4.59 *±* 1.60	0.03*	0.043(−0.017, 0.132)
Urea, (Mean **±** SD), (mg/dl)					
Baseline	32.75 *±* 12.22	31.77 *±* 13.07	30.75 *±* 8.89	0.73	−0.11(−0.016, 0.026)
24 h post PCI	31.13 *±* 13.92	26.67 *±* 13.01	29.42 *±* 9.56	0.24	0.007(−0.016, 0.070)
48 h post PCI	35.24 *±* 14.07	34.18 *±* 12.75	35.96 *±* 8.86	0.79	−0.012(−0.016, 0.020)
Crcl, (Mean **±** SD), (ml/min)					
Baseline	103.88 *±* 30.32	101.93 *±* 24.51	96.47 *±* 24.87	0.41	−0.002(−0.016, 0.052)
48 h post PCI	95.45 *±* 29.19	95.75 *±* 28.71	94.51 *±* 29.56	0.98	−0.015(−0.016, −0.015)
eGFR, (Mean **±** SD), (ml/min/1.73 m2)					
Baseline	75.82 *±* 17.45	78.20 *±* 20.12	75.75 *±* 17.77	0.78	−0.012(−0.016, 0.021)
48 h post PCI	69.51 *±* 18.11	71.95 *±* 21.32	74.25 *±* 23.62	0.59	−0.007(−0.016, 0.037)
Absolute changes (baseline to 48 h) post-PCI	−6.31 *±* 20.35	−6.25 *±* 19.75	−1.5 *±* 21.97	0.468	−0.004(−0.016, 0.047)

^Data are expressed as mean ±SD or Median (Minimum−Maximum), or n (%)^

^TMZ, trimetazidine; Scr, serum creatinine; Crcl, Creatinine clearance; eGFR, estimated glomerular filtration rate^

^For significances * *p*<0.05, ** *P*<0.01, *** *P*<0.001^

### Incidence of CA-AKI among the study groups

**Fig. 2:**
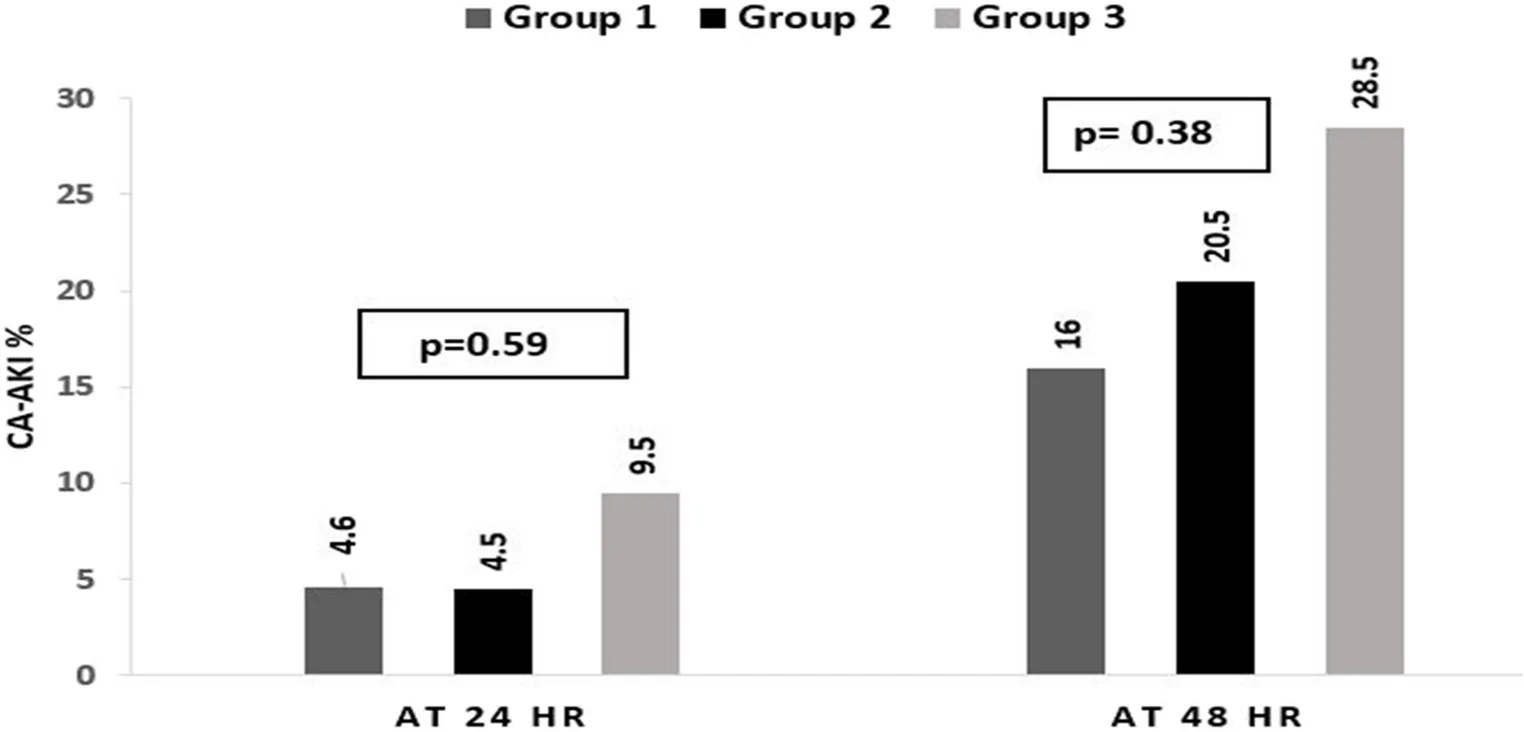
Incidence of CA-AKI at 24 and 48 h among the three study groups

The incidence of CA-AKI was insignificantly lower in Group 1 (IV isotonic saline + TMZ + allopurinol) compared to Group 2 (IV isotonic saline + TMZ) and Group 3 (IV isotonic saline only) at both 24- and 48-hour post-PCI (CA-AKI at 24 h: 2 (4.6%), 2 (4.5%), and 4 (9.52%); at 48 h: 7 (16.28%), 9 (20.45%), and 12 (28.57%) for Groups 1, 2, and 3 respectively; *p* = 0.59 and *p* = 0.38), Fig. 2.

No significant difference was detected between the three groups concerning change in Scr, CrCl, uric acid, and eGFR at 24- and 48-hours post-PCI. All absolute changes at 24- and 48-hours post-PCI in these parameters were non-significant (Table 3).

### ACEF and Mehran scores as predictors of CA-AKI

**Fig. 3:**
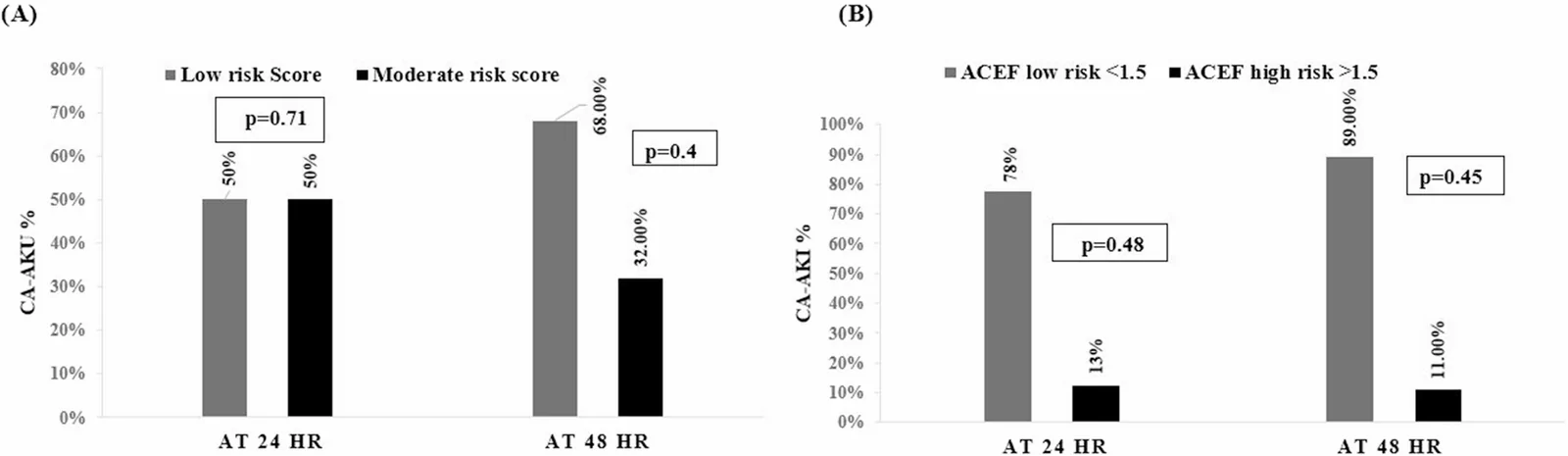
Association between Mehran and ACEF risk categories and the incidence of CA-AKI

Among patients without CA-AKI at 48 h (*n* = 101), 7 (6.9%) were in the highest ACEF tertile, compared to 3 (10.7%) among those who developed CA-AKI (*n* = 28) (*p* = 0.45). Similarly, 41 (40.6%) non-CA-AKI patients and 9 (32.14%) CA-AKI patients were categorized as moderate risk by the Mehran score (*p* = 0.42). Univariate and multivariate Logistic regression analysis at 48 h post-PCI revealed no significant association between ACEF or Mehran scores and the risk of CA-AKI, (Fig. 3). The adjusted odds ratio (aOR) for ACEF score was 1.90 (95% CI: 0.54–6.74), and for Mehran score was 0.95 (95% CI: 0.81–1.12). These findings indicate that neither risk score independently predicted CA-AKI in this low- to moderate-risk cohort. Given the limited clinical significance of early changes, 24-hour outcomes are not reported in detail. A weak positive correlation was observed between the ACEF score and Scr levels at both 24 and 48 h (*r* = 0.21, *p* = 0.017; *r* = 0.20, *p* = 0.024, respectively). In contrast, no significant correlation was found between the Mehran score and Scr levels at either 24–48 h.

### Factors associated with CA-AKI among the study groups

While both CA-AKI at 24-hour and 48-hour were evaluated, the focus on the 48-hour time point for inferential analysis due to its greater clinical relevance and more consistent association with CA-AKI. Due to the non-predictive value of ACEF and Mehran scores and their overlap with individual clinical variables, these scores were not retained in the final multivariable model. In multivariable analysis, older age, higher BMI, and use of ACE inhibitors were significantly associated with increased CA-AKI risk. Diabetic patients appeared to have lower risk, likely influenced by SGLT2 inhibitor use where none of these patients developed CA-AKI. Due to zero cases, SGLT2 users were excluded from the regression model. These findings, though limited by sample size, suggest a protective role for SGLT2 inhibitors. Another finding worth noting is the strong statistical association with baseline Scr, which showed an extremely low odds ratio. This may point to a real effect of baseline kidney function, but it could also reflect issues with how the variable is scaled or how it interacts with other variables in the model. Neither the volume of contrast used, the rate of hydration, nor treatment with TMZ or TMZ + allopurinol significantly influenced CA-AKI risk at 48 h (Table 4).

**Table 4:** Univariate and multivariate logistic regression for 48-hour contrast-induced nephropathy

	Univariate regression			Multivariate regression		
	OR	95% CI	*p*-value	OR	95% CI	*p*-value
Gp = Control			0.383			0.531
Gp = Trimetazidine	0.643	0.238–1.733	0.383	1.015	0.265–3.894	0.983
Ggp = Trimetazidine + Allopurinol	0.486	0.17–1.39	0.178	0.499	0.12–2.079	0.339
Sex = female				0.511	0.144–1.813	0.299
Age				1.081	1.015–1.151	0.016*
BMI				1.164	1.049–1.292	0.004*
DM				0.114	0.029–0.444	0.002*
HTN				3.265	0.72–14.793	0.125
ACE Inhibitors				7.016	1.762–27.933	0.006*
ARBs				2.117	0.473–9.482	0.327
DPP-4 Inhibitors				0.35	0.034–3.616	0.378
Diuretics				0.521	0.1–2.714	0.439
Volume of Hydration ml/hr				1.004	0.981–1.028	0.744
Dye type = Sodium Ioxitalamate				2.112	0.639–6.984	0.221
Dye volume/Vial				0.961	0.779–1.185	0.71
Baseline Serum creatinine				0.001	0–0.066	0.001*
Constant	0.4	0.205–0.782	0.007	0.017	0–2.194	0.1

^For significances * *p*<0.05, ** *P*<0.01, *** *P*<0.001^

### Predictors of 48-Hour increase in serum creatinine

Linear regression analysis was conducted using two models to identify predictors of 48-hour serum creatinine change: one comparing the combination of TMZ + allopurinol versus control (Table 5), and a second comparing TMZ alone versus control (Table 6). In both models, the study treatments did not significantly alter creatinine change. SGLT2 inhibitor use was independently associated with a significantly smaller increase in creatinine in the combination model (β = − 0.261, 95% CI − 0.445 to − 0.077, *p* = 0.007). DPP-4 inhibitors demonstrated a consistent negative association with creatinine rise, reaching statistical significance in the TMZ-only model (β = − 0.195, 95% CI − 0.342 to − 0.048, *p* = 0.012). Diuretic use was associated with a significantly greater creatinine increase in the TMZ-only model (β = 0.2, 95% CI 0.049 to 0.351, *p* = 0.011). Although ACE inhibitor use showed a positive association with creatinine increase in both models, this effect did not reach statistical significance (*p* > 0.05). Baseline serum creatinine remained a strong inverse predictor in both models (*p* < 0.01).

**Table 5:** Linear regression for 48-hour increase in serum creatinine (Combination vs. control)

	Beta	95% CI	*p*-value
(Constant)	0.267	−0.203–0.737	0.271
Combination	−0.058	−0.164–0.048	0.289
Gender	−0.082	−0.192–0.028	0.145
Age	0.003	−0.003–0.009	0.297
BMI	0.006	−0.002–0.014	0.151
Diabetes	0.001	−0.111–0.113	0.991
HTN	0.017	−0.106–0.14	0.788
ACE Inhibitors	0.108	−0.014–0.23	0.087
ARBs	0.024	−0.111–0.159	0.728
DPP-4 Inhibitors	−0.146	−0.299–0.007	0.067
SGLT-2	−0.261	−0.445 - −0.077	0.007**
Diuretics	0.059	−0.09–0.208	0.44
Volume of Hydration ml/hr	−0.001	−0.003–0.001	0.302
Dye type = Sodium Ioxitalamate	0.1	−0.008–0.208	0.071
Dye volume/Vial	−0.001	−0.021–0.019	0.917
Baseline Serum creatinine	−0.48	−0.756 - −0.204	0.001**

^Beta, regression coefficient; For significances * *p*<0.05, ** *P*<0.01, *** *P*<0.001^

**Table 6:** Linear regression for 48-hour increase in serum creatinine (Trimetazidine vs. control)

	Beta	95% CI	*p*-value
(Constant)	0.331	−0.112–0.774	0.148
Trimetazidine	0	−0.102–0.102	0.994
Gender	−0.072	−0.182–0.038	0.209
Age	0.002	−0.002–0.006	0.55
BMI	0.004	−0.004–0.012	0.39
Diabetes	−0.043	−0.161–0.075	0.476
HTN	−0.003	−0.121–0.115	0.962
ACE Inhibitors	0.063	−0.057–0.183	0.3
ARBs	0.023	−0.106–0.152	0.729
DPP-4 Inhibitors	−0.195	−0.342 - −0.048	0.012*
SGLT-2	−0.104	−0.278–0.07	0.248
Diuretics	0.2	0.049–0.351	0.011*
Volume of Hydration ml/hr	−0.001	−0.003–0.001	0.6
Dye type = Sodium Ioxitalamate	0.089	−0.009–0.187	0.08
Dye volume/Vial	−0.005	−0.025–0.015	0.629
Baseline Serum creatinine	−0.397	−0.693 - −0.101	0.011*

^Beta, regression coefficient, For significances * *p*<0.05, ** *P*<0.01, *** *P*<0.001^

To further illustrate these findings, the mean 48-hour serum creatinine change was − 0.04 ± 0.158 mg/dL among SGLT2 inhibitor users versus + 0.096 ± 0.237 mg/dL in non-users, and − 0.05 ± 0.217 mg/dL among DPP-4 inhibitor users versus + 0.098 ± 0.237 mg/dL in non-users. Full regression outputs are presented in Tables 5 and 6.

## Discussion

One of the serious hospital-acquired complications associated with cardiovascular procedures is CA-AKI [7]. All CM agents are cytotoxic, and each agent’s ionic strength, osmolality, or viscosity may have an impact on this [7]. In the general population of patients with stable eGFR ≥ 30 mL/min 1.73 m^2^, prophylaxis against CA-AKI is not advised; nevertheless, in high-risk situations, it may be taken into consideration on an individual basis [36]. While a reduced GFR is a well-established risk factor, other clinical and procedural variables, such as diabetes mellitus, advanced age, volume depletion, hemodynamic instability, and contrast volume, also contribute significantly [7]. Importantly, prior studies have shown that even patients classified as ‘low risk’ based solely on preserved eGFR may still develop CA-AKI, particularly when comorbidities such as diabetes, advanced age, or anemia are present [37–39].This reinforces that eGFR, while a central determinant of risk, does not fully capture the multifactorial nature of CA-AKI susceptibility. To date, studies with positive outcomes of various preventive measures beyond IV hydration have been challenged by meta-analyses and opposing results [19, 23, 40, 41].

To our knowledge, this is the first investigation to assess the combined use of TMZ and allopurinol in elective PCI patients. In the current study, none of the enrolled patients were classified as high-risk for CA-AKI based on either the Mehran or ACEF risk scores. Among patients with low to moderate risk undergoing PCI, the combination of TMZ and allopurinol with IV hydration using normal saline was associated with a non-significant reduction in CA-AKI incidence at 48 h compared with the TMZ-only and hydration-only groups (16.28%, 20.45%, and 28.57%, respectively). While some studies have suggested benefit of using allopurinol or TMZ individually with hydration, our study demonstrated only a non-significant trend with the combination, which should be interpreted with caution and considered hypothesis-generating rather than confirmatory [19, 23, 41].

Previous studies have reported heterogeneous outcomes. For example, Han Fu et al. 2021 [23], found TMZ plus hydration significantly reduced CA-AKI in elderly patients undergoing elective PCI with CrCl < 60 mL/min compared to hydration alone, whereas our younger, lower-risk cohort with preserved eGFR did not show a similar benefit. In that trial, either IOCM or LOCM was used depending on renal function, and CA-AKI occurred in 3.2% of the TMZ group versus 9.7% of controls. In contrast, contrast media selection in the present study was guided by anticipated procedural duration and institutional availability, and both HOCM and LOCM were used. Interestingly, despite the present study’s lower-risk profile, the incidence of CA-AKI across all groups was higher than in the referenced study. This discrepancy could not be clearly attributed to contrast volume, contrast type, or baseline risk factors, and may partly reflect the limited sample size. Importantly, it emphasizes that preserved eGFR alone does not fully capture vulnerability to CA-AKI, and that additional clinical and periprocedural factors—such as advanced age, anemia, diabetes, or unmeasured hemodynamic fluctuations—may have contributed [37–39].

The control group showed a higher incidence of CA-AKI despite receiving a lower contrast volume. This may be due to residual confounding, unmeasured procedural complications, or sample size variation. It may also indicate a possible trend toward benefit with trimetazidine and allopurinol, although the study was not powered to establish statistical significance. Larger studies with systematic collection of procedural and hemodynamic data are warranted to clarify these findings.

Other studies have similarly reported benefits in higher-risk patients. Erol et al. 2013 [19].

evaluated 300 mg of allopurinol 24 h before contrast administration plus hydration versus hydration alone in patients undergoing cardiac catheterization with SCr > 1.1 mg/dL. CA-AKI occurred in 7.5% of controls but in none of the allopurinol group (*p* = 0.013). They concluded that in high-risk patients, allopurinol with hydration may prevent CA-AKI. LOCM (iohexol) was used, with similar contrast volumes in both groups, suggesting that patient risk profile and contrast type may explain differences compared with the present trial.

In the present study, uric acid levels were significantly lower in the TMZ + allopurinol group at baseline and remained so at 24 and 48 h, yet no significant within-group changes were observed. Since all patients had baseline uric acid within the normal range, the expected lowering effect of allopurinol on uric acid may have been attenuated. In normouricemic patients, the magnitude of uric acid reduction with allopurinol is often limited, which may explain the absence of a statistically significant change despite reductions in uric acid observed in prior studies [19, 42]. Risk stratification scores also performed suboptimally. Neither Mehran nor ACEF scores significantly predicted CA-AKI in our cohort. Originally developed in high-risk PCI populations, these tools may have limited predictive value in low- to moderate-risk patients [10, 43, 44]. A weak correlation between ACEF score and serum creatinine was observed at 24–48 h, highlighting the need for refined risk models in such populations.

Conversely, other studies have reported no substantial advantage of adding TMZ or allopurinol to standard hydration [24, 25, 45].Galal, H.et al.** 2020** [24] compared TMZ, allopurinol, and hydration versus hydration alone in elective PCI patients (CrCl > 30 mL/min) and found no significant differences in CA-AKI incidence across groups (25%, 22.5%, and 27.5%,respectively). The type of CM was not specified. The absence of observed benefit in these trials may partly reflect methodological limitations, such as lack of formal risk stratification and variable contrast exposure. Overall, the similarity of these findings to the current study suggests that in patients with preserved renal function (eGFR > 60 mL/min), neither TMZ nor allopurinol provides significant protection against CA-AKI. Furthermore, in this subgroup, other common risk factors appeared to have limited impact on CA-AKI incidence, highlighting the difficulty of demonstrating treatment effects in low- to moderate-risk populations.

Moreover, two meta-analyses assessed the efficacy of allopurinol in the prevention of CA-AKI [17, 46].Bellos I et al. 2019 [17] concluded that allopurinol may preserve renal function after contrast exposure and recommended future large-scale trials exploring its combination with other renoprotective agents. Similarly, Mansoor et al. 2021 [46] found that allopurinol (100–600 mg) significantly reduced CA-AKI incidence compared with hydration alone, with consistent benefit observed in sensitivity analyses restricted to 300 mg dosing.

Additionally, two more meta-analyses have shown that TMZ is effective in avoiding CA-AKI [26, 47].Heshmatzadeh Behzadi et al. 2021 [47] reported a threefold reduction in CA-AKI incidence among high-risk patients undergoing angioplasty or angiography receiving TMZ in addition to standard hydration. More recently, Nair et al. 2024 [26] confirmed TMZ’s nephroprotective effect after cardiac intervention across subgroups, including patients with baseline renal impairment.

One particularly interesting finding was the apparent benefit of SGLT2 inhibitors. None of the patients on these agents developed CA-AKI, and they showed smaller increases in Scr at 48 h. While encouraging, this observation is based on a very small subgroup and must be interpreted cautiously. Nevertheless, it is consistent with emerging evidence, including a recent meta-analysis reporting a 63% reduction in CA-AKI risk with SGLT2 inhibitors in diabetic patients undergoing coronary angiography or PCI [48] Similarly, a multicenter registry study conducted on patients with type 2 diabetes mellitus showed significantly lower CA-AKI incidence in SGLT2 inhibitor users after primary PCI [49].

Additionally, patients receiving DPP-4 inhibitors showed smaller increases in serum creatinine. Experimental and clinical studies suggest these agents may confer nephroprotection by reducing inflammation, oxidative stress, and fibrosis, promoting natriuresis, attenuating fibrosis and apoptosis in diabetic conditions, providing protection in both diabetic and non-diabetic CKD models, and modulating the gut–renal axis through both GLP-1-dependent and independent mechanisms [50, 51] [52, 53].

However, given the small number of patients and the inability to exclude confounding by diabetic status or background therapies, the results regarding SGLT2 inhibitors and DPP-4 inhibitors should be considered exploratory and hypothesis-generating rather than definitive.

In summary, previous studies have shown that either TMZ or allopurinol alone can significantly reduce the incidence of CA-AKI in high-risk patients with baseline GFR < 60 mL/min/1.73 m² [19, 23, 26, 41, 46, 47]. However, consistent with the findings of the current study, neither agent demonstrated a significant protective effect against CA-AKI in patients with normal renal function, regardless of comorbidities.

### Study limitations

This study has several limitations. First, it was conducted at a single center, which may restrict the generalizability of the findings. Second, the relatively small sample size may have reduced the statistical power to detect significant differences between groups. The sample size calculation was based on Bodagh et al. (2019), which assumed a large absolute risk reduction (26%), whereas the observed reduction (~ 12%) was smaller, likely decreasing power and increasing the risk of a type II error. Third, most participants were classified as low to moderate risk for CA-AKI based on the ACEF and Mehran scores, with baseline eGFR > 60 mL/min/1.73 m². This predominance, reflecting the elective PCI population, may have contributed to the low CA-AKI incidence and limited ability to detect between-group differences. Additionally, CA-AKI was defined according to the ESUR (1999) criteria, consistent with Xu et al. (2016). Re-evaluation of the study data using KDIGO thresholds (≥ 0.3 mg/dL or ≥ 1.5–1.9 times baseline increases within 48 h) yielded a comparable incidence of CA-AKI, confirming that applying KDIGO criteria would not have altered the study’s conclusions [2, 34, 35]. Fourth, patients with left ventricular systolic dysfunction (EF < 50%) did not receive routine IV hydration according to institutional protocol due to concerns about fluid overload. Although alternative preventive measures were applied in line with ESUR recommendations, this individualized approach may limit comparability with other studies [4]. Fifth, procedural complications and hemodynamic parameters during PCI were not systematically collected; factors such as periprocedural hypotension or embolization could therefore represent residual confounders [54, 55]. Finally, both HOCM and LOCM were used, and their distribution was not strictly uniform across groups. Although HOCM was used in only a small minority of patients, this may have introduced residual confounding. Taken together, these limitations suggest that the findings should be interpreted cautiously as hypothesis-generating and validated in larger, multicenter trials enrolling patients with a broad spectrum of CA-AKI risk factors.

## Conclusion

The combination of trimetazidine and allopurinol did not significantly reduce the incidence of CA-AKI compared with trimetazidine alone or hydration alone in patients with low to moderate risk undergoing elective PCI. Nonetheless, a favorable, though non-significant, trend toward lower CA-AKI incidence was observed with the combination therapy. Associations between DPP-4 and SGLT-2 inhibitor use and smaller increases in serum creatinine suggest potential nephroprotective effects, but these findings remain exploratory and require confirmation in larger prospective studies. The limited predictive accuracy of the ACEF and Mehran scores underscores the need for improved risk-stratification models that incorporate additional clinical and medication-related factors.

Overall, these results should be interpreted as hypothesis-generating and provide a rationale for larger, multicenter trials to confirm the potential renoprotective benefit of trimetazidine combined with allopurinol.

## Data Availability

The data underlying this article will be shared on reasonable request to the corresponding author.
